# In-Vitro Antibacterial and Anti-Inflammatory Effects of Surfactin-Loaded Nanoparticles for Periodontitis Treatment

**DOI:** 10.3390/nano11020356

**Published:** 2021-02-01

**Authors:** Athira Johnson, Fanbin Kong, Song Miao, Sabu Thomas, Sabah Ansar, Zwe-Ling Kong

**Affiliations:** 1 Department of Food Science, National Taiwan Ocean University, Keelung 20224, Taiwan; athirajohnson07@gmail.com; 2 Department of Food Science and Technology, University of Georgia, Athens, GA 30602, USA; fkong@uga.edu; 3 Teagasc Food Research Centre, Moorepark, Fermoy, Co., Cork P61 C996, Ireland; song.miao@teagasc.ie; 4 School of Energy Studies and School of Chemical Sciences, Mahatma Gandhi University, Priyadarshini Hills P.O, Kottayam, Kerala 686560, India; sabuthomas@mgu.ac.in; 5 Department of Clinical Laboratory Sciences, College of Applied Medical Sciences, King Saud University, P.O. Box 10219, Riyadh 11433, Saudi Arabia; sansar@ksu.edu.sa

**Keywords:** periodontitis, nanoparticle, surfactin, antibacterial, anti-inflammatory

## Abstract

Periodontitis is an inflammatory disease associated with biofilm formation and gingival recession. The practice of nanotechnology in the clinical field is increased overtime due to its potential advantages in drug delivery applications. Nanoparticles can deliver drugs into the targeted area with high efficiency and cause less damages to the tissues. In this study, we investigated the antibacterial and anti-inflammatory properties of surfactin-loaded κ-carrageenan oligosaccharides linked cellulose nanofibers (CO-CNF) nanoparticles. Three types of surfactin-loaded nanoparticles were prepared based on the increasing concentration of surfactin such as 50SNPs (50 mg surfactin-loaded CO-CNF nanoparticles), 100SNPs (100 mg surfactin-loaded CO-CNF nanoparticles), and 200SNPs (200 mg surfactin-loaded CO-CNF nanoparticles). The results showed that the nanoparticles inhibited the growth of *Fusobacterium nucleatum* and *Pseudomonas aeruginosa*. The reduction in biofilm formation and metabolic activity of the bacteria were confirmed by crystal violet and MTT assay, respectively. Besides, an increase in oxidative stress was also observed in bacteria. Furthermore, anti-inflammatory effects of surfactin-loaded CO-CNF nanoparticles was observed in lipopolysaccharide (LPS)-stimulated human gingival fibroblast (HGF) cells. A decrease in the production of reactive oxygen species (ROS), transcription factor, and cytokines were observed in the presence of nanoparticles. Collectively, these observations supported the use of surfactin-loaded CO-CNF as a potential candidate for periodontitis management.

## 1. Introduction

Periodontitis is an oral disease characterized by gingival bleeding, tissue destruction, gingival pocket formation, and tooth loss. Literature mentioned that periodontitis affects about 20–50% of the global population [1,2]. It is understood that severe periodontitis is considered as the sixth most prevalent oral disease worldwide [3]. This disease can be facilitated by various risk factors. Literature mentioned that risk factors for periodontitis are classified into modifiable (cigarette smoking, diabetes mellitus, socioeconomic and demographic variables, psychosocial variables, and stress, etc.) and non-modifiable risk factors (age and genetics) [4]. However, periodontitis is mainly developed due to the formation of biofilms by microorganisms, and these biofilms are the organized bacterial communities and it leads to local inflammation in the gingiva [5]. Species such as *Aggregatibacter actinomycetemcomitans*, *Porphyromonas gingivalis*, *Eikenella corrodens*, *F. nucleatum*, *Treponema denticola*, *Streptococcus mutans*, and *Campylobacter rectus* are well-known to cause periodontitis [6,7]. The organized biofilms are called dental plaque and they are either attached to the tooth surface or other microorganisms. Later, the biofilms form under the gingival surface due to biofilm maturation and subsequent dysbiosis. Besides, organic acids are produced after sugar metabolism by bacterial plaque and it causes pH reduction and tooth demineralization [8]. As a result, the periodontal tissues are damaged due to the host immune reaction that arises in response to microbes [9]. Both the innate and adaptive immune system plays a crucial role in the progression of periodontitis. The inflammatory pathway begins with the infiltration of polymorphonuclear (PMN) cells like neutrophils and natural killer (NK) cells. Complement component C3 is also activated as a result of the host immune response. It is noted that toll-like receptors (TLR)-2 and TLR-4 are highly responsive to bacterial products including lipopolysaccharides (LPS). Cytokines and chemokines are released by subsequent reactions and cause damage to the periodontal tissues [10,11]. Along with this, the reactive oxygen species (ROS) production and lipid peroxidation are also increased under periodontitis conditions. It causes fibroblast DNA damage and affects polymorphonuclear leukocytes by increasing hydrogen peroxide production. The ROS production is induced by polymorphonuclear leukocytes upon bacterial antigen stimulation via oxidative burst [2]. It is noted that periodontitis enhances the risk of some other diseases such as diabetes, rheumatoid arthritis, obesity, cancer, and cardiovascular diseases, etc. [9,12]. So, it is necessary to take care of this disease. Conventionally, it can be treated by various methods such as scaling, root planing, antibiotic administration, and surgery [4]. Sometimes, these methods are limited due to root damage, high cost, side effects, pain, and reduced efficacy [4,13]. Therefore, novel therapeutic approaches are needed to overcome the limitations of current therapies.

Nanotechnology-based drug delivery systems combined with biological and physical science can develop nanostructures and this kind of structures have active participation in the pharmaceutical field. Nanomaterials have a size range between 1 and 100 nm and exhibit good mechanical and chemical properties [14]. It is noted that biopolymers are highly suitable for nanoparticles preparation due to their biocompatibility, biodegradability, and low immunogenicity [15]. Nanocellulose is a biopolymer obtained from cellulose by mechanical, chemical, and enzymatic treatments. It is categorized into three forms such as cellulose nanocrystals (CNC), cellulose nanofibers (CNF), and bacterial cellulose (BC) [16]. CNF is renowned for its high surface area to volume ratio, porosity, flexibility, strength, and biocompatibility, etc. [17]. CNF have a size range between 20 and 100 nm in width and >10,000 nm in length. Nanocellulose is highly reactive due to its hydroxyl groups. However, the surface functionalization of the nanocellulose helps to attain the desirable properties that increase the effectiveness of nanocellulose in drug delivery [18]. In this study, CNF was modified with κ-carrageenan oligosaccharide (CO). Carrageenan is a water-soluble polysaccharide extracted from red seaweed [19]. κ-Carrageenan is a sulfated linear polysaccharide of D-galactose and 3,6-anhydro-d-galactose [20]. The medical applications of this compound with regard to immune response, anti-tumor activity, and stem cell proliferation are being examined [21]. Our previous study reported that the crystallinity, swelling properties, and degradation temperature of CNF were improved after modifying with CO [13].

Surfactin is incorporated into CO-CNF to evaluate the antimicrobial and anti-inflammatory properties. It is an amphiphilic biosurfactant largely extracted from the *Bacillus subtilis* species. It is known that surfactin exhibits various pharmacological activities such as anti-inflammatory, antibacterial, anti-mycoplasmal, antifungal, antiviral, anticancer, and thrombolytic activities. It has a polar amino acid head and a hydrocarbon ring [22]. Surfactin has a cyclic heptapeptide composed of L-Glu, L-Leu, D-Leu, L-Val, L-Asp, D-Leu, and L-Leu and it is linked to a β-hydroxy fatty acid group [23]. Surfactin produce antimicrobial activity by disrupting the bacterial membrane via altering the hydrophobicity of the bacterial surface and the subsequent inability of the bacteria to attach the solid surfaces [24,25]. Apart from that, it also interacts with the biological membrane via chelating mono and divalent cations, altering membrane permeability, and changing membrane solubility. The penetration is facilitated by the conformational changes of the peptide cycle [26]. As a result, membrane integrity will be lost and cause cellular leakage [27]. The inhibitory effect of surfactin on *S. mutans* was reported before [28]. Furthermore, synergeistic antimicrobial effects of surfactin and ampicillin was reported against *P. aeruginosa* ATCC 27853 [29]. Besides, Huang et al. (2018) developed surfactin-based nanoparticles loaded with doxorubicin to overcome multidrug resistance in cancers [30]. The literature stated that surfactin reduce inflammatory condition via attenuating the activation of nuclear factor (NF)-κB involved with TLR-4 in LPS-induced macrophages [31].

We have previously explored the antibacterial effect of surfactin-loaded CO-CNF nanoparticles against two pathogens such as *P. gingivalis* and *S. mutans*. This material has a good release profile and entrapment efficiency. Moreover, it significantly reduced the biofilm (bacterial layer) formation and metabolic activity of *P. gingivalis* and *S. mutans* [13]. In this paper, we extended the study by evaluating the antimicrobial properties of surfactin-loaded CO-CNF nanoparticles in two other bacteria such as *F. nucleatum* and *P. aeruginosa. F. nucleatum* is an anaerobic gram-negative bacterium that facilitates biofilm formation due to its adhesive property. It also binds to various mammalian cells and invades epithelial and endothelial cells [32]. It belongs to the Fusobacteriaceae family and it acts as a bridging organism to connect other microorganisms and also interact with them. They can co-aggregate with initial and late colonizers. This may be achieved because of its long rod-shaped structure. Besides, the bacterium uses its surface molecules for the attachment. *F. nucleatum* interacts with host cells via adhesin expression and induces the production of cytokines such as IL-6 and IL-8. It stimulates the production of tumor necrosis factor (TNF)-α by activating the NF-κB pathway by influencing myeloid cells [10,33]. *P. aeruginosa* is a major respiratory pathogen that is found in the oral cavity and it acts as a link between pulmonary disease and periodontitis. Studies showed the presence of *P. aeruginosa* in the oral microbiota of chronic periodontitis. They are capable of forming biofilm by producing extracellular enzymes and toxins [34]. Another study also reported the presence of *P. aeruginosa* in the subgingival biofilm [35]. Under periodontitis conditions, the growth and attachment of respiratory pathogens increased and the inhalation of oral pathogens into the lung cause lung disorders. *P. aeruginosa* can cause pneumonia and cystic fibrosis [36,37]. In addition to this, we explored the anti-inflammatory properties of nanoparticles under LPS-induced inflammatory conditions in human gingival fibroblast (HGF) cells. They are the most abundant cells in the gingiva and exhibit cytokines production in the presence of LPS without LPS tolerance [38]. We hypothesized that the proposed material has better antibacterial and anti-inflammatory activities to prevent periodontitis inflammation. The antimicrobial used in this study was surfactin. Three types of surfactin-loaded nanoparticles were prepared based on surfactin concentration such as 50SNPs (50 mg surfactin-loaded CO-CNF nanoparticles), 100SNPs (100 mg surfactin-loaded CO-CNF nanoparticles), and 200SNPs (200 mg surfactin-loaded CO-CNF nanoparticles). It is a potent antimicrobial agent and assumed that the incorporation of surfactin into the carrier (CO-CNF) will produce beneficial effects over periodontitis. Apart from this, materials such as CNF and CO are well-known to develop drug delivery systems. So, the current study evaluated the surfactin loaded CO-CNF nanoparticles for the in vitro periodontitis management. The antibacterial activities were evaluated in *F. nucleatum* and *P. aeruginosa* and the anti-inflammatory activities were evaluated in LPS-induced HGF cells.

## 2. Materials and Methods

### 2.1. Materials

Human gingival fibroblast cell line was purchased from Blossom Biotechnologies Inc., Taipei, Taiwan. *Pseudomonas aeruginosa* (ATCC 9027) and *Fusobacterium nucleatum* (ATCC 10953) were obtained from Bioresource Collection and Research Center, Hsinchu, Taiwan. poly-L-lysine and fibroblast medium were purchased from Blossom Biotechnologies Inc., Taipei, Taiwan. κ- carrageenan oligosaccharide was obtained from Dah Chung Trading Co., Ltd., Taipei, Taiwan. Sodium hydroxide (purity ≥ 97.0%), trichloroacetic acid (purity ≥ 99.0%), N,N′-methylenebisacrylamide (purity 99%), and potassium bromide (purity 100%) were obtained from Sigma-Aldrich, St. Louis, MO, USA. Soybean curd residue was obtained from Kuang Chuan Dairy Co., Ltd., Taipei, Taiwan. Methanol (purity > 99.9%) was obtained from Honeywell Burdick & Jackson, Muskegon, MI, USA. Hydrochloric acid (36.5–38.0%) was got from Panreac AppliChem, Darmstadt, Germany. Crystal violet (≥90.0% anhydrous basis), 3-(4,5-dimethylthiazol-2-yl)-2,5-diphenyl tetrazolium bromide (purity 100%), thiobarbituric acid (purity ≥ 98%), dimethyl sulfoxide (purity ≥ 99.9%), tryptic soy broth, surfactin (purity ≥ 98.0%), and acridine orange (purity ≥ 98%) were purchased from Sigma-Aldrich, St. Louis, MO, USA. Doxycycline was purchased from Swiss Pharmaceuticals Co., Ltd., Tainan, Taiwan. Nuclear factor-kappa B was purchased from Asia Bioscience Co. Ltd., Taipei, Taiwan. Prostaglandin E2 ELISA kit and interleukin-6 ELISA kit were purchased from Taiclone Biotech Corp., Taipei, Taiwan.

### 2.2. Methods

#### 2.2.1. Preparation of Surfactin-Loaded CO-CNF Nanoparticles

**Figure 1:**
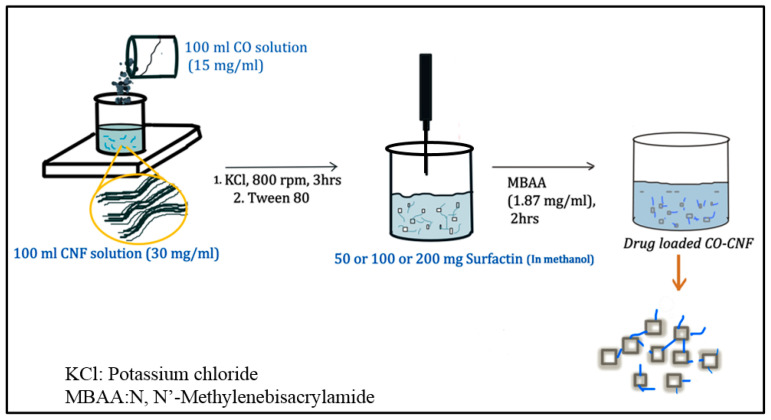
Schematic representation of surfactin-loaded nanoparticles preparation.

The detailed procedures for particle preparation and characterization were reported in our previously reported work [13]. Briefly, 100 mL of CO (1 g in 100 mL) solution was mixed with 100 mL of CNF solution (30 mg/ ml) and stirred at 80 °C for 10 min. Later, potassium chloride (KCl) was added as a cross-linker and stirred at 80 °C. After 3 h, the particles were collected by centrifugation, and then sonicated (HOYU Ultrasonic 250, Taipei, Taiwan), and freeze-dried. The surfactin-loaded CO-CNF nanoparticles were prepared by the emulsion method. Three types of nanoparticles were prepared in this study such as 50SNPs (50 mg surfactin-loaded CO-CNF nanoparticles), 100SNPs (100 mg surfactin-loaded CO-CNF nanoparticles), and 200SNPs (200 mg surfactin-loaded CO-CNF nanoparticles) based on the concentration of surfactin. 200 µL of Tween 80 was added to 50 mL of CO-CNF solution and stirred for 1h at 60 °C. Afterwards, surfactin (50/100/200 mg in 2 mL methanol) solution was taken and CO-CNF solution was added to it. As a stabilizer, 8 mL of N,N′-methylenebisacrylamide (MBAA) (1.87 mg/mL) was added. After 2 h of reaction, the obtained particles were sonicated and free-dried. A schematic representation of surfactin loaded nanoparticles preparation was given in Figure 1.

#### 2.2.2. Bacteria Culture and Agar Ditch Plate Method

*F. nucleatum* (ATCC 10953) and *P. aeruginosa* (ATCC 9027) were cultured at 37 °C in tryptic soy broth (TSB). The *F. nucleatum* was cultured in anaerobic conditions (anaerobic jar under anaerobic gas pack sachet). The gas pack was opened and placed in the jar together with bacteria culture. The jar was then kept inside the incubator at 37 °C. The purity was determined by subculturing the bacteria and checking the colony morphology. The Agar ditch plate method was used to determine the antimicrobial property of the samples. Samples were dispersed in water and doxycycline (10 mg/mL) was selected as a positive control throughout the study. 10 µL bacterial (1 × 10^7^ CFU/mL) solution was coated on agar plates (TSB) and 100 µL of doxycycline, surfactin (10 mg/mL), and nanoparticles (20 mg/mL) were added to wells that made on the agar that already coated by the respective bacteria. After incubation (24 h, 37 °C), the zone of inhibition was evaluated [13].

#### 2.2.3. Minimum Bactericidal Concentration (MBC) and Minimum Inhibitory Concentration (MIC) Evaluation

For determining the MIC and MBC, samples were serially diluted from their initial concentration (20 mg/mL). In total, 120 μL of bacterial culture (1 × 10^5^ CFUs/mL) were added to a 96-well plate and incubated (37 °C) with 80 µL of each sample. The *F. nucleatum* plate was sealed with paraffin films to avoid contamination. The MIC value was determined by evaluating the visible growth of microorganisms. For determining the MBC value, 100 μL of culture broth was taken from the well and swabbed into an agar plate. After 24 h of incubation, the MBC value was measured. The experiment was repeated three times and the values of MIC and MBC were given in percentage [39].

#### 2.2.4. Inhibition of Bacterial Layer Formation

The wells were washed three times with water after 24 h of incubation of sample (80 µL) and 120 µL of bacterial species (1 × 10^7^ CFU/mL) and later, 0.1% (*v*/*v*) crystal violet solution (200 µL/mL) was added (kept for 1 h). Afterward, 200 μL of 95% (*v*/*v*) ethanol was added to solubilize the crystal violet. The experiment was repeated three times and absorbance was determined at 595 nm (ELISA Reader, Thermo Fisher 1510, Dreieich, Germany). Inhibition of bacterial layer formation is determined by the following equation:

Inhibition of bacterial layer formation (%)

(1) [1−(Absorbance of cells treated with drugs/absorbance of non treated control cells)]×100

#### 2.2.5. Bacterial Viability (MTT Assay) and Malondialdehyde (MDA) Content Evaluation

The viability of bacteria (1 × 10^7^ CFU/mL) after sample treatment (80 µL) was checked by using 3-(4,5-dimethylthiazol-2-yl)-2,5-diphenyl tetrazolium bromide (MTT) assay. The medium was removed after 24 h of incubation and refilled with 100 µL of MTT (1mg/mL) for 4 h. Then, the MTT solution was removed and 200 µL of dimethyl sulfoxide (DMSO) was added. The optical density was checked at 570 nm. MDA assay was conducted to evaluate the oxidative stress in bacteria. The solution containing treated samples and 200 µL of MDA reagent were placed in a water bath (Water Bath, BUCHI 461, Zürich, Switzerland) at 100 °C for 15 min. The MDA reagent containing a mixture of 47 mL water, 1 mL HCl, 7.2 g trichloroacetic acid, and 0.18 g thiobarbituric acid. The standard was 1,1,3,3-tetramethoxypropane. After that, n-butanol (300 µL) was added and centrifuged (1500 × g, 10 min). The absorbance of the supernatant was measured at 532 nm [40]. Bacterial viability and MDA content were calculated from equations (2) and (3).

(2) $$\mathrm{Bacterial}\;\mathrm{viability}\;(\%)\;=\frac{Absorbance\;of\;the\;sample-Absorbance\;of\;the\;blank}{Absorbance\;of\;the\;control-Absorbance\;of\;the\;blank}\;\times 100$$

(3) $$\mathrm{MDA}\;\mathrm{level}\;(\mathrm{nmol}/\mathrm{mL})=\;\frac{Absorbance\;of\;sample\;at\;532\;\mathrm{nm}-Absorbance\;of\;blank\;at\;532\;\mathrm{nm}}{Absorbance\;of\;standard\;at\;532\;\mathrm{nm}-Absorbance\;of\;blank\;at\;532\;\mathrm{nm}}\;\times 5$$

#### 2.2.6. Acridine Orange (AO) Assay

The bacterial death is confirmed by acridine orange assay. Bacteria (350 µL) were treated with nanoparticles (150 µL) for 24 h and then stained with acridine orange solution (1 mg/mL). The cells were imaged under a microscope after washing the cells by centrifugation (3000 rpm, 3 min) [41].

#### 2.2.7. Cell Viability Evaluation

Human gingival fibroblast (HGF) cells were cultured at 37 °C in a fibroblast medium containing fibroblast growth supplement (5 mL), fetal bovine serum (10 mL), and penicillin/streptomycin (5 mL). The cell line was purchased from Blossom Biotechnologies Inc., Taipei, Taiwan (isolated from human gingiva). The cells are known as the main structural part of the gingiva and were spindle-shaped. MTT assay was performed to analyze the toxicity of the samples. The MTT is converted into formazan crystals by dehydrogenases occurring in the mitochondria of living cells. Literature also mentioned that some reducing agents and enzymes from other organelles and endoplasmic reticulum are also responsible for this conversion [42]. The cell number was adjusted to 1 × 10^4^ cells/well in a 96-well plate and incubated for 24 h. Afterwards, 100 µL of samples (50 µg/mL) was added and incubated for another 24 h. Then, MTT reagent (100 µL) was added and kept in a CO_2_ incubator for 4 h. Finally, 200 µL of DMSO solution was and the optical density was determined at 570 nm.

#### 2.2.8. Superoxide Anion Production

After pre-treatment (200 µL of samples (50 µg/mL) and 100 µL of LPS (1 µg/mL)), the cells (2 × 10^5^ cells/well) were incubated for 24 h in a CO_2_ incubator. The cells were collected by centrifugation (800× *g* for 15 min) and 300 µL of NBT solution was added. After centrifugation (1500× *g*, 15 min), 200 µL DMSO was added to the cells and the optical density was determined at 570 nm. The percentage of NBT reduction inhibition percentage was evaluated by Equation (4).

(4) $$\mathrm{Percentage}\;\mathrm{of}\;\mathrm{NBT}\;\mathrm{reduction}\;\mathrm{inhibition}\;(\%)\;=\;\frac{A\;control-A\;sample}{A\;control}\;\times 100$$

#### 2.2.9. Evaluation of Nitric Oxide and Malondialdehyde Production

The cells (1 × 10^4^ cells/well) were pre-treated (20 µL of nanoparticles (50 µg/mL) and 10 µL of LPS (1 µg/mL)). After 24 h of incubation, the cell culture medium (50 µL) was taken and added to a 96-well plate containing 50 µL of Griess reagent. The plate was incubated for 10 min and the optical density was analyzed at 540 nm. Sodium nitrate (50 µL) was the standard. The NO production was evaluated by plotting the standard curve. The MDA analysis was conducted after pre-treatment. The procedures were the same as in Section 2.2.5.

#### 2.2.10. Cytokines Productions

Cytokines productions were evaluated using ELISA kits. Briefly, after pre-treatment with samples and LPS, the supernatant was collected, and the level of PGE2, NF-κB and IL-6, and PGE2 were determined. All procedures were conducted according to the manufacturer’s instructions. For NF-κB determination, the solution was removed after incubating 100 µL standard/samples for 1 h. Afterward, detection reagent A (100 µL) and 100 µL detection reagent B were added and incubated for 1h and 30 min, respectively. After washing (5 times), the plates were incubated with 90 µL substrate solution for 15 min. Later, 50 µL of stop solution was added and measured the absorbance at 540 nm. For IL-6 and PGE2 determination, samples/standard and 50 µL biotinylated detection Ab working solution were added to the plate and kept in an incubator for 45 min at 37 °C. Later, the plate was incubated with 100 µL of HRP conjugate working solution for 30 min. After washing, the plate was incubated with 90 µL of substrate solution for 15 min. Finally, a 50 µL stop solution was added and the optical density measured at 540 nm.

### 2.3. Statistical Analysis

Origin Pro 2018 SR1 b9.5.1.195 (OriginLab Corporation, Northampton, MA 01060, USA) software was used to analyze the data. The results were given as mean ± standard deviation (S.D.). Significant differences were determined by one-way ANOVA with *p* < 0.05 considered as significantly different followed by Tukey multiple comparison tests.

## 3. Results

### 3.1. Determination of MIC and MBC

The obtained MIC and MIC values of samples were given in Table 1. CO-CNF and 50SNPs have no minimum inhibitory effects on *F. nucleatum* up to its 120% concentration. Both 100SNPs and 200SNPs shared the same MIC value (100%). It was noted that only 200SNPs (120%) showed MBC value for the *F. nucleatum*. The MIC value of CO-CNF and 50SNPs were similar for *P. aeruginosa* but only 50SNPs showed MBC value at its maximum concentration. 100SNPs and 200SNPs inhibited the visible growth of *P. aeruginosa* at 80% and 70% concentrations, respectively. The MBC values of 100SNPs and 200SNPs were 100% and 90%, respectively. It was understood that *P. aeruginosa* was more sensitive towards nanoparticles rather than *F. nucleatum*.

The results were given in percentages of the sample concentration (20 mg/mL). MIC: minimum inhibitory concentration; MBC: minimum bactericidal concentration; N: No value obtained; CO-CNF: κ-carrageenan oligosaccharides linked cellulose nanofibers; 50SNPs: 50 mg surfactin-loaded CO-CNF nanoparticles; 100SNPs: 100 mg surfactin-loaded CO-CNF nanoparticles; 200SNPs: 200 mg surfactin-loaded CO-CNF nanoparticles.

### 3.2. Agar Well Diffusion Method

**Figure 2:**
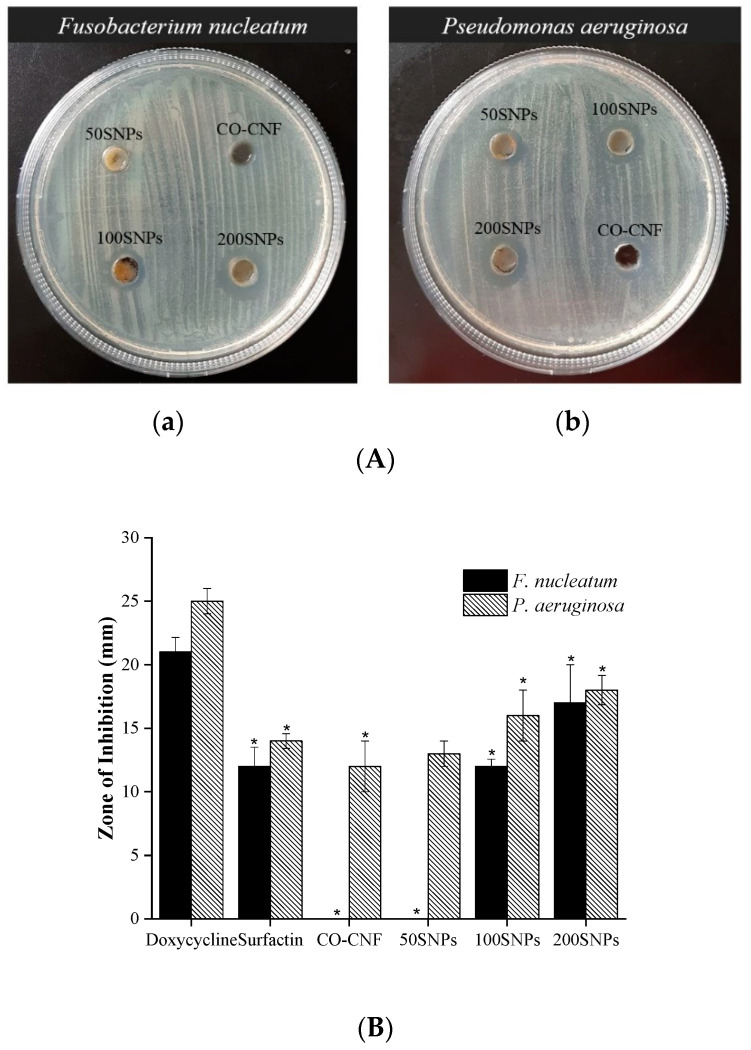
(**A**) Determination of antimicrobial properties of nanoparticles against (**a**) *Fusobacterium nucleatum* and (**b**) *Pseudomonas aeruginosa*. (**B**) Zone of inhibition of nanoparticles. Data are expressed as mean ± SD (*n* = 3). The asterisk (*) represents a significant difference (*p* < 0.05) from the positive control (doxycycline) analyzed by Tukey’s test. CO-CNF: κ-carrageenan oligosaccharides linked cellulose nanofibers; 50SNPs: 50 mg surfactin-loaded CO-CNF nanoparticles; 100SNPs: 100 mg surfactin-loaded CO-CNF nanoparticles; 200S NPs: 200 mg surfactin-loaded CO-CNF nanoparticles.

Figure 2A,B showed the inhibitory effect of nanoparticles after 24 h of incubation. It was observed that only 100SNPs (12 mm) and 200SNPs (17 mm) have a zone of inhibitions (ZI) against the *F. nucleatum*. However, it was lower than the doxycycline (21 mm) (positive control). Surfactin and 100SNPs shared similar ZI values. 50SNPs and CO-CNF have no ZI towards the *F. nucleatum*. All the groups showed a significant ZI against *P. aeruginosa*. A Higher ZI value was seen in doxycycline (25 mm) followed by 200SNPs (18 mm). The ZI values of CO-CNF, 100SNPs, and 200SNPs were 12, 13, 16 mm, respectively. The ZI value of surfactin was higher than 50SNPs but lower than 100SNPs.

### 3.3. Biofilm (Bacterial Layer) Formation Inhibition

**Figure 3:**
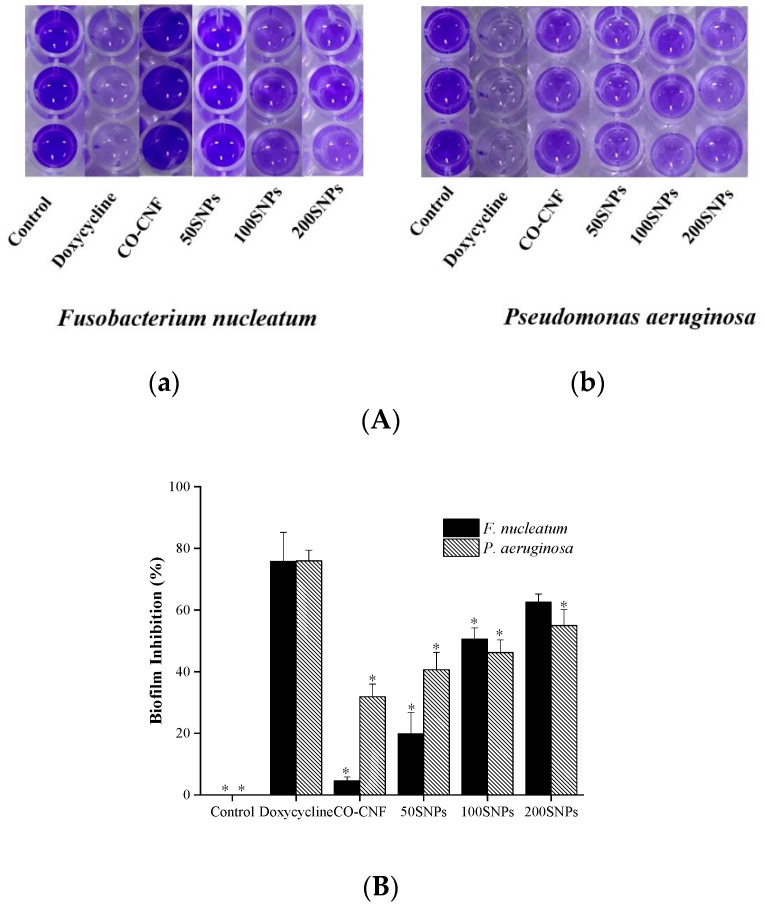
(**A**)Visualization of biofilm formation by (**a**) *Fusobacterium nucleatum* and (**b**) *Pseudomonas aeruginosa* and (**B**) quantification of biofilm inhibition in *Fusobacterium nucleatum* and *Pseudomonas aeruginosa*. Data are expressed as mean ± S.D. (*n* = 3). The asterisk (*) represent a significant difference (*p* < 0.05) from positive control (doxycycline) analyzed by Tukey’s test. CO-CNF: κ-carrageenan oligosaccharides linked cellulose nanofibers; 50SNPs: 50 mg surfactin-loaded CO-CNF nanoparticles; 100SNPs: 100 mg surfactin-loaded CO-CNF nanoparticles; 200SNPs: 200 mg surfactin-loaded CO-CNF nanoparticles.

Hundreds of bacterial species and extracellular matrix constitute the biofilms in the oral cavity. The percentage of biofilm inhibition against bacteria were given in Figure 3. As shown in Figure 3 the highest biofilm inhibition for *F. nucleatum* was observed in doxycycline-treated groups followed by 200SNPs. More than 40% of biofilm inhibition was seen in 100SNPs. There was no significant difference between CO-CNF and the control group. The inhibition capacity of 50SNPs lies between CO-CNF and 100SNPs. The biofilm inhibition of nanoparticles against *P. aeruginosa* was also evaluated. It was noted that doxycycline prevents the highest percentage of biofilm formation followed by 200SNPs. There was no significant difference between 200SNPs and 100SNPs. The situation was also similar in the case of CO-CNF and 50SNPs, however the highest percentage of biofilm inhibition was observed in 50SNPs.

### 3.4. Metabolic Activity of Bacteria

**Figure 4:**
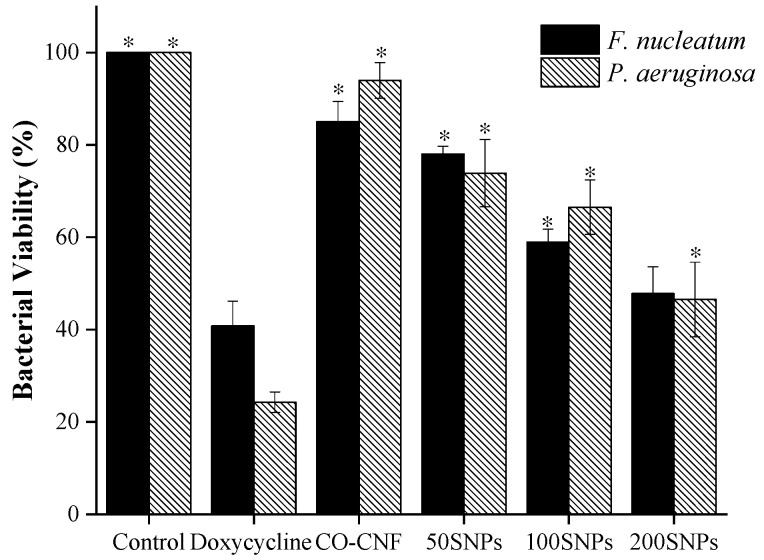
Effects of nanoparticles on the metabolic activity of *Fusobacterium nucleatum* and *Pseudomonas aeruginosa*. Data are expressed as mean ± S.D. (*n* = 3). The asterisk (*) represent significant differences (*p* < 0.05) from positive control (doxycycline) analyzed by Tukey’s test. CO-CNF: κ-carrageenan oligosaccharides linked cellulose nanofibers; 50SNPs: 50 mg surfactin-loaded CO-CNF nanoparticles; 100SNPs: 100 mg surfactin-loaded CO-CNF nanoparticles; 200S NPs: 200 mg surfactin-loaded CO-CNF nanoparticles.

The viability of bacteria after being treated with nanoparticles was given in Figure 4. The highest percentage of viability of the *F. nucleatum* was seen in the control group followed by CO-CNF and 50SNPs (Figure 4). The viability of the *F. nucleatum* in the 200SNPs group was higher than doxycycline but both of them were not significantly different. Like *F. nucleatum*, The *P. aeruginosa* viability was increased in the control group together with CO-CNF (Figure 4). Less than 80% viability was observed in surfactin loaded nanoparticles, especially in 200SNPs. However, it was significantly higher when compared to doxycycline.

### 3.5. Malondialdehyde (MDA) Production

**Figure 5:**
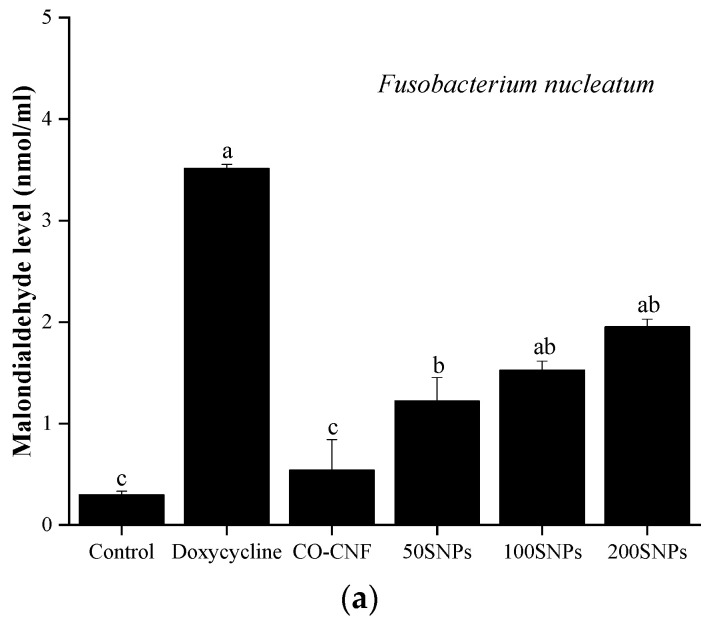

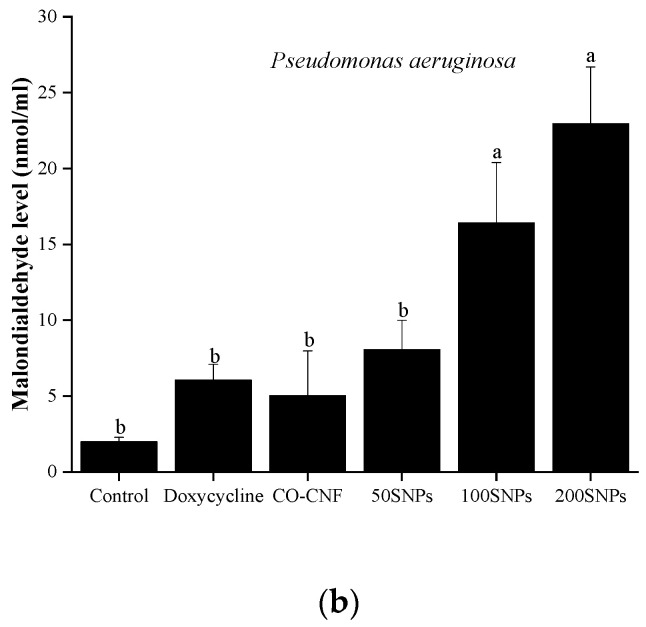
Effects of nanoparticles on the malondialdehyde level of (**a**) *Fusobacterium nucleatum* and (**b**) *Pseudomonas aeruginosa*. Data are expressed as mean ± S.D. (*n* = 3). The values with different letters (a–c) represent significantly different (*p* < 0.05) as analyzed by Tukey’s test. CO-CNF: κ-carrageenan oligosaccharides linked cellulose nanofibers; 50SNPs: 50 mg surfactin-loaded CO-CNF nanoparticles; 100SNPs: 100 mg surfactin-loaded CO-CNF nanoparticles; 200SNPs: 200 mg surfactin-loaded CO-CNF nanoparticles.

MDA is known as the marker of lipid peroxidation and it is associated with ROS generation [43]. Figure 5a showed that MDA production was highly increased by doxycycline treatment in the *F. nucleatum*. The MDA level was slightly increased from CO-CNF to 200SNPs group. There was no significant difference was observed between 100SNPs and 200SNPs. Both in control and CO-CNF groups, the MDA production was decreased, and it was less than 50SNPs group. The MDA production in the *P. aeruginosa* group was highly increased by 200SNPs followed by 100SNPs treatments. The lowest MDA level was seen in the control group, but it was not significantly different from doxycycline, CO-CNF, and 50SNPs.

### 3.6. Acridine Orange Assay

**Figure 6:**
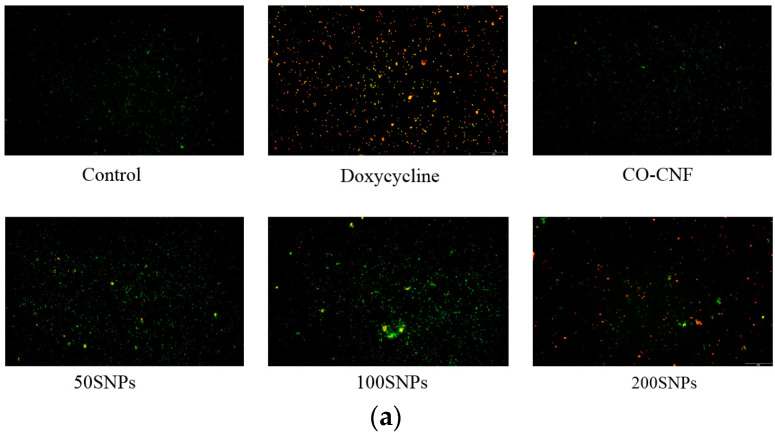

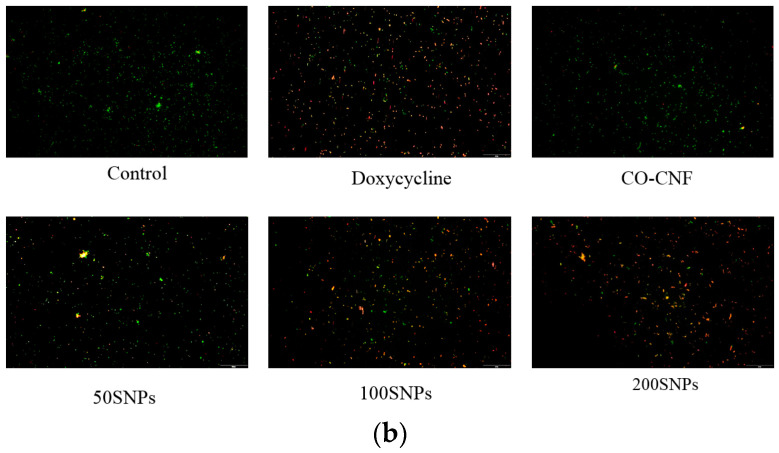
Acridine orange (AO) assay of (**a**) *Fusobacterium nucleatum* and (**b**) *Pseudomonas aeruginosa* treated with different samples. CO-CNF: κ-carrageenan oligosaccharides linked cellulose nanofibers; 50SNPs: 50 mg surfactin-loaded CO-CNF nanoparticles; 100SNPs: 100 mg surfactin-loaded CO-CNF nanoparticles; 200SNPs: 200 mg surfactin-loaded CO-CNF nanoparticles.

The viability of bacteria after being treated with nanoparticles was confirmed by AO assay (Figure 6). The green color indicating viable cells and the red color indicating the dead cells. It was understood that 200SNPs was stronger against *F. nucleatum* along with doxycycline. Only a few dead cells were observed in 50SNPs and 100SNPs. More viable *P. aeruginosa* was seen in both CO-CNF and the control groups. 50SNPs and 100SNPs have dead cells but it was less than 200SNPs and doxycycline.

### 3.7. Cell Viability

**Figure 7:**
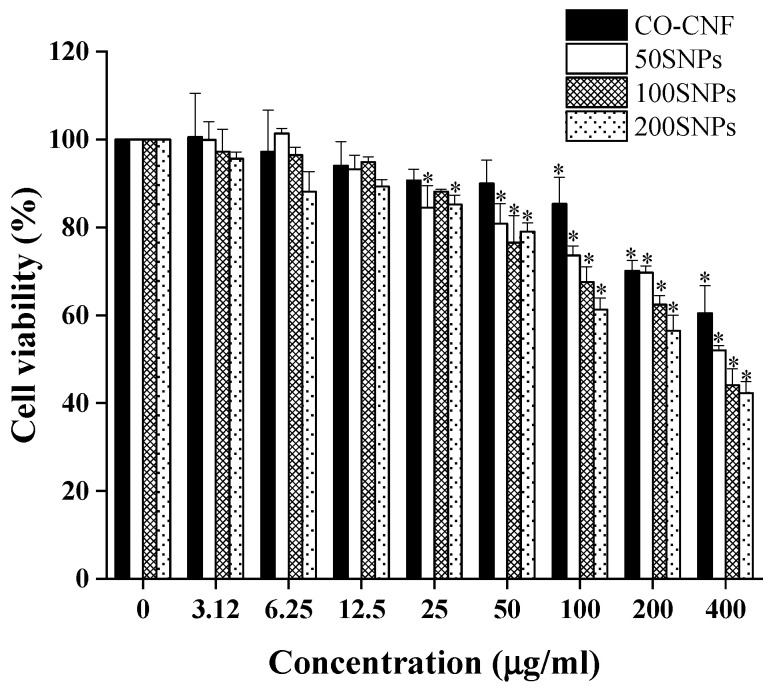
Cell viability of human gingival fibroblast cells (1 × 10^4^ cells/well) in the presence of nanoparticles after 24 h. Data are expressed as mean ± S.D. (*n* = 3). The asterisk (*) indicated the significant differences of the groups from control at *p* < 0.05 analyzed by the Tukey test. CO-CNF: κ-carrageenan oligosaccharides linked cellulose nanofibers; 50SNPs: 50 mg surfactin-loaded CO-CNF nanoparticles; 100SNPs: 100 mg surfactin-loaded CO-CNF nanoparticles; 200SNPs: 200 mg surfactin-loaded CO-CNF nanoparticles.

The toxicity of nanoparticles was checked in HGF cell lines (Figure 7). It was understood that up to 25 µg/mL, all nanoparticles exhibited more than 80 percent of cell viability. The viability of CO-CNF and 50SNPs treated cells was dropped below 80% from 200 and 100 µg/mL. Both 50SNPs and 200SNPs showed a significant difference from the control group at 25 µg/mL concentration. However, CO-CNF and 100SNPs showed a significant difference from the control group at 100 and 50 µg/mL concentration. Furthermore, it was understood that the cell viability was dropped with an increasing concentration of surfactin.

### 3.8. Oxidative Stress

**Figure 8:**
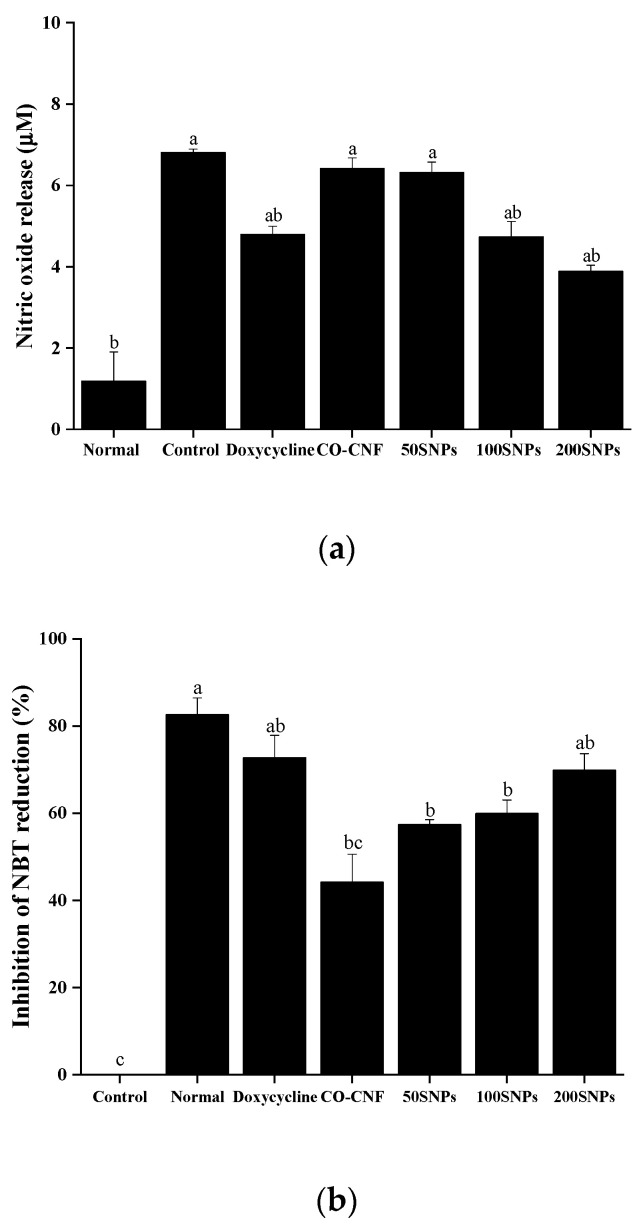

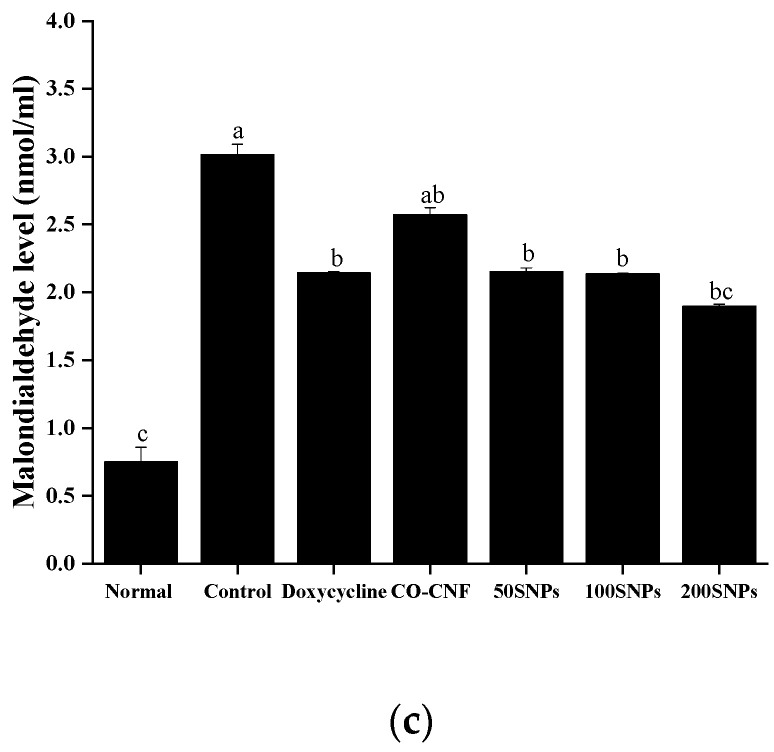
(**a**) nitric oxide production, (**b**) inhibition of nitroblue tetrazolium reduction, and (**c**) malondialdehyde level in nanoparticles treated lipopolysaccharide (LPS) stimulated human gingival fibroblast cells. The cells (1 × 10^4^ cells/well) were pre-treated with 20 µL of samples (50 µg/mL) and 10 µL of LPS (1 µg/mL). Data are expressed as mean ± S.D. *(n* = 3). The letters (a–c) indicated the significant differences of the groups at *p* < 0.05 analyzed by the Tukey test. CO-CNF: κ-carrageenan oligosaccharides linked cellulose nanofibers; 50SNPs: 50 mg surfactin-loaded CO-CNF nanoparticles; 100SNPs: 100 mg surfactin-loaded CO-CNF nanoparticles; 200SNPs: 200 mg surfactin-loaded CO-CNF nanoparticles.

The imbalance between the production of ROS and antioxidant levels leads to oxidative stress and associated inflammation [44]. NO, superoxide anion, and MDA generation in nanoparticles treated LPS-stimulated HGF cells were given in Figure 8. NO is a free radical and the level of NO is increased under periodontitis condition by the activity of bacterial LPS and pro-inflammatory mediators [45]. As shown in Figure 8a, the NO production was higher in the control group and lower in the normal group. NO production in both CO-CNF and 50SNPs was not significantly different from the control group. NO production was reduced in doxycycline and 100SNPs. However, it was higher when compared to 200SNPs. Superoxide anion is another ROS responsible for oxidative stress within the tissues. The production of superoxide anion was highly reduced in doxycycline and 200SNPs, along with the normal group (Figure 8b). Both 50SNPs and 100SNPs were not statistically different, and the inhibition percentage was higher than CO-CNF. MDA is produced as the result of the peroxidation of polyunsaturated fatty acids and it is recognized as the marker of oxidative stress [44]. As shown in Figure 8c, the MDA level was highly increased in the control group and reduced in the normal group. The MDA level in three groups such as doxycycline, 50SNPs, and 100SNPs was not statistically different. Besides, it was lower than the CO-CNF group. Furthermore, it was observed that the MDA production was highly reduced in 200SNPs when compared to other groups.

### 3.9. Anti-Inflammatory Properties

**Figure 9:**
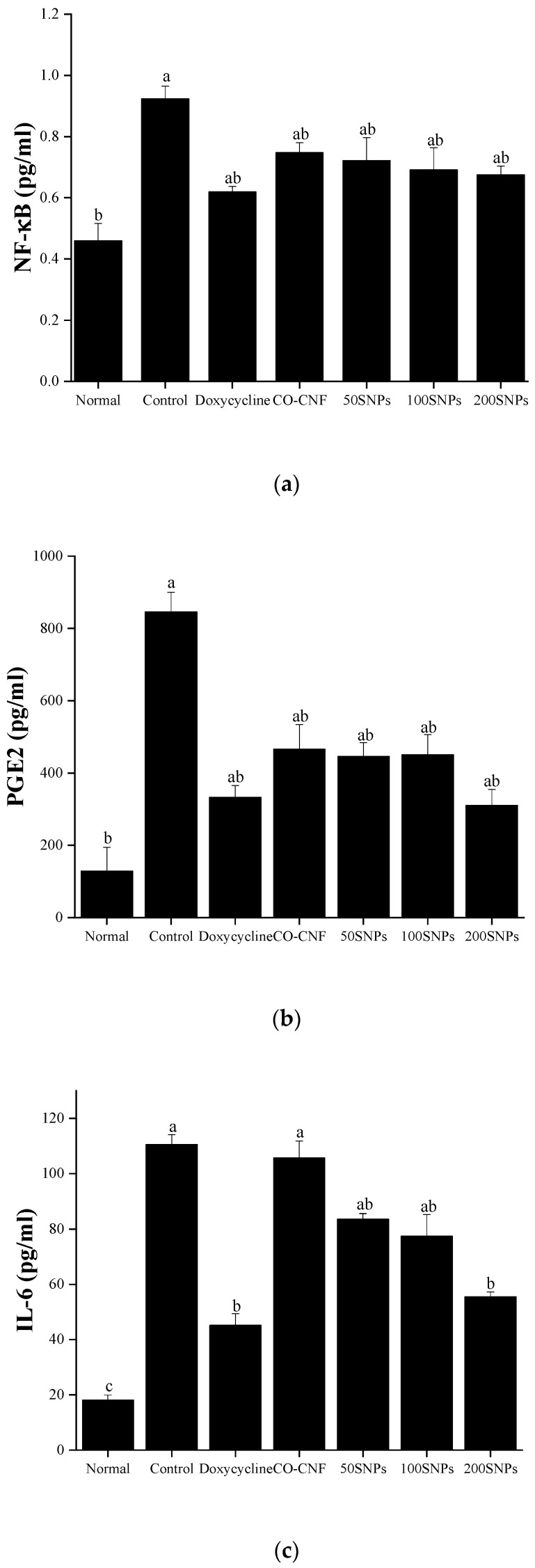
Level of (**a**) nuclear factor (NF)-κB, (**b**) prostaglandin E2, and (**c**) interleukin-6 in nanoparticle treated lipopolysaccharide (LPS)-stimulated cells. The cells (1 × 10^4^ cells/well) were pre-treated with 20 µL of samples (50 µg/mL) and 10 µL of LPS (1 µg/mL). Data are expressed as mean ± S.D. (*n* = 3). The letters (a–c) indicated the significant differences of the groups at *p* < 0.05 analyzed by the Tukey test. Abbreviations: CO-CNF: κ-carrageenan oligosaccharides linked cellulose nanofibers; 50SNPs: 50 mg surfactin-loaded CO-CNF nanoparticles; 100SNPs: 100 mg surfactin-loaded CO-CNF nanoparticles; 200SNPs: 200 mg surfactin-loaded CO-CNF nanoparticles.

Periodontitis is an inflammatory disease associated with a high level of pro-inflammatory cytokines. The levels of NF-κB, PGE2, and IL-6 after treatment with nanoparticles were given in Figure 9. It was noted that NF-κB expression was increased in the control group and highly reduced in the normal group (Figure 9a). A slight reduction in NF-κB expression was observed upon the increasing concentration of surfactin but they were not significantly different. Doxycycline showed a notable reduction in NF-κB expression, but it was not statistically different except for control and normal groups. Like NF-κB, the PGE2 level was also higher in the control group (Figure 9b). CO-CNF, 50SNPs, and 100SNPs shared almost equal PGE2 level, but it was significantly higher than doxycycline and 200SNPs. PGE2 levels in both doxycycline and 200SNPs were not statistically different but doxycycline contained a higher level of PGE2 than 200SNPs. The amount of IL-6 was given in Figure 9c. A higher level of IL-6 was observed in the control group followed by CO-CNF. IL-6 level was reduced in both 50SNPs and 100SNPs but it was higher than 200SNPs. IL-6 level in doxycycline was not statistically different when compared to 200SNPs but it is lesser than 200SNPs.

## 4. Discussion

Periodontitis is a chronic inflammatory disease that leads to the loss of gingiva and alveolar bone. This may happen due to the formation of bacterial biofilms and the production of inflammatory mediators [3]. Literature mentioned that about 700 species of bacteria are residing in the oral cavity and the bacterial colonies are attached to the surface and each other by producing a sticky extracellular polymeric substance [46]. This biofilm will be matured to form plaque and cause inflammation. The inflammation begins with the infiltration of neutrophils into the affected area [47]. Later, TLR activates innate host response and leads to the activation of several transcription factors including NF-κB and activator protein 1 (AP-1) through the mitogen-activated protein kinase (MAK) cascade. This results in the production of cytokines such as IL-1, IL-6, and TNF-α and chemokines [48].

The combination of nanotechnology and biology created a new scientific area called nanomedicine or nanobiotechnology. It is noted that the targeted and safe delivery of drugs into the gingival area can be achieved by using nanoparticles. The current study is focused on CNF- and CO-based drug delivery system loaded with surfactin. The CO-CNF nanoparticles were prepared in the presence of KCl (cross-linker). Surfactin was added to the above solution and the surfactin-loaded CO-CNF was obtained by emulsification. Here, the CO-CNF is acting as a drug carrier for the potential delivery of surfactin [13]. The literature stated that nanocellulose has been used as filler in many dental materials. It is a reinforcing agent and can improve the mechanical properties and are able to form network structure [49]. The use of carrageenan in the pharmaceutical field is increased due to its biocompatibility and consolidation behavior. It will help to improve the drug formulation properties including prolonged drug release [50]. Surfactin is a biosurfactant that has large application in the clinical field. Previously published articles reported the inhibition of *Klebsiella pnemoniae*, *Salmonella typhimurium* NCTC 74, *Staphylococcus aureus* ATCC 6538, *and Escherichia coli* NCTC 10418 in presence of surfactin [51]. Another study also reported the inhibition of the production of pro-inflammatory cytokines by surfactin via blocking the activation of *P. gingivalis* LPS-triggered NF-κB [52]. A decrease in the production of IFN-γ, IL-6, iNOS, and NO by surfactin in LPS-activated macrophages were also reported [31]. Another study also confirmed the reduction of pro-inflammatory cytokines in the presence of surfactin [53].

The current study is focused on the antimicrobial and anti-inflammatory properties of surfactin loaded CO-CNF nanoparticles. CO-CNF particles were prepared in the presence of KCl. Our previous study reported that this material has an average size of 330 nm and the zeta potential was about −42 mV. It was understood that the addition of CO improved the swelling ability and degradation temperature of the CNF. Surfactin-loaded CO-CNF was a chain-like structure and exhibited controlled drug release. It has good water holding capacity and prevented the growth of periodontal pathogens such as *S. mutans* and *P. gingivalis* [13]. In this paper, we extended our study by evaluating the antimicrobial activity in another two pathogens named *F. nucleatum* and *P. aeruginosa*. Besides, we analyzed the anti-inflammatory properties in LPS-stimulated HGF cells. MIC and MBC values are determined to identify the inhibitory concentration of each bacteria. MIC is the lowest concentration that will inhibit the visible growth of a microorganism and MBC is the minimum concentration that prevents the growth of bacteria [54]. Dilution methods are commonly used for determining the MIC and MBC values [55]. Here, the samples were diluted from its original concentration (20 mg/mL). Therefore, the MIC and MBC values were expressed in percentages. Our results suggested that a higher concentration of 200SNPs can inhibit the growth of *F. nucleatum* and 100SNPs was also effective. As compared to *F. nucleatum*, *P. aeruginosa* was more sensitive to the nanoparticles, especially 200SNPs. Along with surfactin-loaded nanoparticle, CO-CNF also prevented the visible growth of *P. aeruginosa*. Therefore, it was noted that higher concentration nanoparticles can inhibit the growth of periodontal pathogens. A previous study reported that the MIC range (mg/L) of amoxicillin towards *F. nucleatum* was between <0.016–4, while the MIC range (mg/L) of tetracycline was 0.016–0.5. The percentage of the susceptibility of tetracycline and amoxicillin towards *A. actinomycetemcomitans* was 99.2% and 100%, respectively [56,57]. Another study has shown that tobramycin and colistin exhibited 72% and 92% susceptibility towards *P. aeruginosa* [58]. In this study, it was observed that the optimum concentration for MIC and MBC is depending upon the concentration of surfactin.

The Agar disc diffusion method is a simple and low-cost assay, known as one of the fundamental techniques to identify the sensitivity of bacteria towards certain drugs. During this assay, the antimicrobials are diffused into the agar and inhibit the growth and germination of bacteria [55]. The inhibition zone was determined with the agar well plate method. It is understood that the susceptibility of the bacteria is closely related to the inhibition zone [59]. Our results showed that only 100SNPs and 200SNPs were strong enough to inhibit the growth of *F. nucleatum*. However, *P. aeruginosa* was more sensitive to all the nanoparticles. Literature mentioned that antibiotics resistance to microorganisms is increasing each year. Therefore, effective drug formulations are required [60]. The antimicrobial activity of the nanoparticle is strongly based on the concentration of surfactin. Surfactin is a surface-active biomolecule that has low toxicity, biodegradability, and can survive in extreme pH and temperature conditions [51]. It was noted that surfactin produces antimicrobial activity by leakage and lysis of lipid membranes of the targeted organisms [61]. However, the nanocarrier provides a suitable environment for the effective delivery of therapeutic agents. They increase the rate of dissolution, promote the interaction between bacterial membranes, and enhance solubility. Because of the high surface area to volume ratio (small size), they can facilitate the interaction and contacts [62]. It was supported by a recently published paper, in which the antimicrobial activity of glutathione-stabilized silver nanoparticles against *F. nucleatum* was reported. The MBC value of this nanoparticle for *F. nucleatum* was ≥98.50 µg/mL [63].

Microbial biofilms are the association of microbial communities embedded in a complex matrix consisting of organic polymers and saliva. Bacteria are stuck to each other and then adhered to the surface [64]. Literature mentioned that this bacterial attachment is influenced by the formation of pellicle via the binding saliva to the enamel [65]. Usually, the biofilm is formed by autoaggregation and coaggregation. Biofilms stimulate cross-feeding, nutrients uptake, and remove harmful metabolic products. They can create an appropriate physiochemical environment and exhibit quorum sensing for their growth. In addition to this, it protects from toxic substances, host defense mechanisms, and other competing microorganisms [66]. So, the reduction in biofilm formation is an important criterion for periodontitis prevention. The current study examined the bacterial layer formation of *F. nucleatum* and *P. aeruginosa* in the presence of nanoparticles (Figure 3). It was observed that the biofilm formation of both bacteria was highly reduced in the doxycycline and 200SNPs groups. Bacteria use surface adhesion and biofilm formation for their survival. Literature mentioned that the antiadhesive property of surfactin is due to the electrostatic repulsion between bacteria and the surfactin [67]. It was also noted that the biosurfactants can alter the bacterial surface hydrophobicity [68]. This type of antimicrobial agent stimulates membrane permeability via damaging the cell membrane. It affects the formation of flagella and alters the hydrophobicity of the membrane. This will contribute to the anti-adhesive property of the biosurfactants [69].

The metabolic state of the bacteria can be influenced by the presence of the antibacterial agent. The action of therapeutic gradually reduced metabolic activity and finally resulted in bacterial cell death. Here, the metabolic activity of bacteria was analyzed by MTT assay. The viable cells reduce the MTT into formazan crystals [70]. As shown in Figure 4, the metabolic activity of *F. nucleatum* and *P. aeruginosa* were reduced in the presence of nanoparticles, especially 200SNPs. Lipid peroxidation is induced by the attack of ROS on unsaturated lipids and can lead to the formation of MDA [43]. Lipid peroxidation is thought to be the major reason for oxidative stress and subsequent cell death. As shown in Figure 5, an increase in MDA production in both bacteria indicating that the nanoparticles can cause bacterial lysis by inducing oxidative stress. The toxicity of nanoparticles on bacteria was confirmed by AO assay. The metabolic state of the bacteria can be understood by the green and red fluorescent emissions. Acridine orange is a cationic dye capable of binding to negatively charged nucleotides. DNA will interact with AO by intercalation while RNA will interact by ionic interactions and dye stacking [71]. DNA fragmentation is a marker for cell death [72]. So, the live/death cells can be visible via AO assay. As shown in Figure 6, nanoparticles were able to cause cell death of both bacteria in its higher concentration. Literature suggested the delivery of antibiotics by using nanocellulose based drug delivery systems. Durable antibacterial properties of allicin were reported when it was conjugated with NCC. The largest holding capacity and sustained release are obtained due to the 3D network structure [73]. Along with surfactin, the antibacterial activity of CO was also reported against *S. aureus*, *E.coli*, and *Penicillium citrinum*, etc. [74].

The cell viability of the nanoparticles was analyzed by MTT assay. It is known as the gold standard for analyzing cell viability. The viability of the cells can be determined by understanding the functionality of mitochondria. The major characteristic of periodontitis is the inflammatory condition in the gingiva. HGF are the most abundant cells in the gingiva and they produce pro-inflammatory cytokines in the presence of LPS [38]. HGF exhibit a strong immunomodulatory role in response to stress and diseases [75]. Our studies have shown that surfactin-loaded nanoparticles maintained more than 80% of human gingival fibroblast cell viability up to 25 µg/mL. Thus, it was understood that the proposed nanoparticles are less toxic to the cells. Our previous study also evaluated the toxicity of surfactin loaded CO-CNF in RAW 267.4 macrophage cells. All groups showed more than 80% viability up to 100 µg/mL concentration [13]. Thus, the results indicate that this material is less toxic to the cells.

Periodontitis is an inflammatory disease characterized by gingival inflammation and alveolar bone loss. ROS can oxidize a variety of biomolecules and finally result in cell death because of oxidative stress [76,77]. Literature mentioned that the inflammation and tissue destruction is due to the excess ROS content in gingival crevicular fluid in association with low antioxidant levels [77]. Superoxide anion (O_2_^•−^), hydroxyl radical (OH•), hydrogen peroxide (H_2_O_2_), and hypochlorous acid (HOCl) are some of the ROS that are highly active to induce inflammation [78]. They play a crucial role in autophagy by inducing NF-κB, JNK, and inflammasome activation [79]. Nitric oxide is generated by the catalysis of nitric oxide synthase. Previous literature stated that NO facilitates the periodontal disease by regulating the action of LPS and cytokines [80]. NBT is a water-soluble compound capable of reacting with cellular superoxide ions and form formazan derivative. Thus, it can understand the content of ROS [81]. Literature mentioned that the reduction in oxygen leads to the formation of superoxide anion and it is then dismutated to form hydrogen peroxide. This hydrogen peroxide can penetrate the cell membrane and generate hydroxyl radicals [82]. This byproduct is known as the central determinant of oral polymicrobial synergy and can trigger defensive inflammatory responses. The higher production of ROS causes changes in the structural integrity of the cells [79]. Lipid peroxidation is the oxidation of polyunsaturated fatty acid caused by free radical-mediated chain reactions [83]. It was known that MDA is a marker for oxidative status in the biological system [84]. Our studies showed that surfactin-loaded nanoparticles can reduce ROS production, thereby preventing the cells from undergoing oxidative stress.

The local inflammatory process by bacteria will lead to potential tissue damage and tooth loss. This process is triggered by the infiltration of neutrophils, natural killer cells (NK) cells, and granulocytes. Later, pro-inflammatory mediators such as TNF-α, IL-1, interferon-γ (IFN-γ), IL-4, IL-10, and transforming growth factor β (TGF-β) [11]. Literature mentioned that the expression of pro-inflammatory cytokines may lead to the activation of NF-κB and this in turn causes the production of inflammatory mediators such as prostaglandins and MMP [85]. PGE2 is an arachidonic acid-derived inflammatory mediator synthesized by immune cells, fibroblasts, and other resident gingival cells [86]. IL-6 is an important cytokine known to stimulate bone resorption and T cell differentiation [87]. Our results showed that nanoparticles can reduce the production of cytokines levels. The previous study also stated that surfactin downregulates the production of pro-inflammatory cytokines by attenuating the activation of NF-κB [31]. The basic physical mechanism behind the antimicrobial activity is the destruction of the biofilm formation and subsequent reduction in the metabolic activity. It was noted that the production of ROS and cytokines were reduced in LPS-stimulated HGF cells. Thus, the results indicated that surfactin-loaded CO-CNF nanoparticle is potentially effective to reduce bacterial biofilm formation and subsequent inflammation. The biopolymer-based drug delivery systems are more suitable for periodontal therapy because of its bioadhesive and biodegradable properties. Efficient delivery of drugs and biocompatibility are the key terms for developing a drug carrier system. Osorio et al., (2016) developed a PolymP-nActive nanoparticles loaded with zinc/or calcium. This study was focused on the calcium phosphate deposition [88]. However, this study was more focused on the mineralization rather than antibacterial properties. Anti-inflammatory properties of minocycline loaded chitosan nanoparticles were reported. The successful internalization of nanoparticles and reduction in the production of cytokines were evident this study [89]. In this study we focused on both antibacterial and anti-inflammatory properties of surfactin-loaded nanoparticles. Our results show that the proposed biomaterial reduced the biofilm formation and metabolic activity of the bacteria. Besides, it reduced the production of ROS and cytokines in LPS-induced human gingival fibroblast cells.

## 5. Conclusions

The demand for the treatment for periodontitis is increased every day due to its prevalence. Periodontitis is an inflammatory disease initiated by bacterial biofilm formation and progressed by inflammation. The treatment based on nanotechnology is getting a lot of attention due to its efficiency and rare side effects. In the present study, we have evaluated the antibacterial and anti-inflammatory properties of surfactin-loaded CO-CNF nanoparticles. The antibacterial properties were evaluated against a periodontal pathogen *F. nucleatum* and an oral bacteria *P. aeruginosa*. Our results show that the nanoparticles, especially 200SNPs, reduced the bacterial viability by inducing oxidative stress, and the inhibition of bacterial biofilm was also observed in the presence of nanoparticles. Furthermore, the anti-inflammatory properties were evaluated in LPS-stimulated human gingival fibroblast cells. The results indicated that the nanoparticles can reduce ROS and cytokine production under an inflammatory condition. Therefore, it can be used as a suitable drug delivery system for periodontitis treatment. However, further studies are required to evaluate the complete efficacy of the nanoparticles. In that sense, the animal clinical studies can proceed in the future based on this study.

## Figures and Tables

**Table 1:** Evaluation of the Minimum Inhibitory Concentration (MIC) and Minimum Bactericidal Concentration (MBC) of nanoparticles.

Microorganisms	MIC (%)				MBC (%)			
	CO-CNF	50SNPs	100SNPs	200SNPs	CO-CNF	50SNPs	100NPs	200SNPs
*Fusobacterium nucleatum*	N	N	100	100	N	N	N	120
*Pseudomonas aeruginosa*	110	110	80	70	N	120	100	90

## Data Availability

The data presented in this study are available on request from the corresponding author.

## References

[1] Nazir M.A. (2017). Prevalence of periodontal disease, its association with systemic diseases and prevention. Int. J. Health Sci..

[2] Tomofuji T., Irie K., Sanbe T., Azuma T., Ekuni D., Tamaki N., Yamamoto T., Morita M. (2009). Periodontitis and increase in circulating oxidative stress. Jpn. Dent. Sci. Rev..

[3] Tonetti M.S., Jepsen S., Jin L., Otomo-Corgel J. (2017). Impact of the global burden of periodontal diseases on health, nutrition and wellbeing of mankind: A call for global action. J. Clin. Periodontol..

[4] Kinane D.F., Stathopoulou P.G., Papapanou P.N. (2017). Periodontal diseases. Nat. Rev. Dis. Primers.

[5] Hasan A., Palmer R.M. (2014). A clinical guide to periodontology: Pathology of periodontal disease. Br. Dent. J..

[6] Mombelli A. (2018). Microbial colonization of the periodontal pocket and its significance for periodontal therapy. Periodontology.

[7] Popova C., Dosseva-Panova V., Panov V. (2013). Microbiology of Periodontal Diseases. A Review. Biotechnol. Biotechnol. Equip..

[8] Bui F.Q., Almeida-da-Silva C.L.C., Huynh B., Trinh A., Liu J., Woodward J., Asadi H., Ojcius D.M. (2019). Association between periodontal pathogens and systemic disease. Biomed. J..

[9] Naiff P.F., Carneiro V.M.A., Guimarães M.d.C.M., Bezerra A.C.B., Oliveira M.S., Couto S.C.P., Alves É.A.R., Kückelhaus S.A.S., Muniz-Junqueira M.I. (2020). Mechanical Periodontal Therapy Recovered the Phagocytic Function of Monocytes in Periodontitis. Int. J. Dent..

[10] Kriebel K., Hieke C., Müller-Hilke B., Nakata M., Kreikemeyer B. (2018). Oral Biofilms from Symbiotic to Pathogenic Interactions and Associated Disease-Connection of Periodontitis and Rheumatic Arthritis by Peptidylarginine Deiminase. Front. Microbiol..

[11] Hoare A., Soto C., Rojas-Celis V., Bravo D. (2019). Chronic Inflammation as a Link between Periodontitis and Carcinogenesis. Mediat. Inflamm..

[12] Winning L., Linden G.J. (2015). Periodontitis and systemic disease. BDJ Team.

[13] Johnson A., He J.-L., Kong F., Huang Y.-C., Thomas S., Lin H.-T.V., Kong Z.-L. (2020). Surfactin-Loaded ĸ-Carrageenan Oligosaccharides Entangled Cellulose Nanofibers as a Versatile Vehicle Against Periodontal Pathogens. Int. J. Nanomed..

[14] Patra J.K., Das G., Fraceto L.F., Campos E.V.R., Rodriguez-Torres M.d.P., Acosta-Torres L.S., Diaz-Torres L.A., Grillo R., Swamy M.K., Sharma S. (2018). Nano based drug delivery systems: Recent developments and future prospects. J. Nanobiotechnol..

[15] Nitta S.K., Numata K. (2013). Biopolymer-based nanoparticles for drug/gene delivery and tissue engineering. Int. J. Mol. Sci.

[16] Salimi S., Sotudeh-Gharebagh R., Zarghami R., Chan S.Y., Yuen K.H. (2019). Production of Nanocellulose and Its Applications in Drug Delivery: A Critical Review. ACS Sustain. Chem. Eng..

[17] Bhandari J., Mishra H., Mishra P.K., Wimmer R., Ahmad F.J., Talegaonkar S. (2017). Cellulose nanofiber aerogel as a promising biomaterial for customized oral drug delivery. Int J. Nanomed..

[18] Trache D., Tarchoun A.F., Derradji M., Hamidon T.S., Masruchin N., Brosse N., Hussin M.H. (2020). Nanocellulose: From Fundamentals to Advanced Applications. Front. Chem..

[19] Al-Baarri A., Legowo A., Rizqiati H., Widayat W., Septianingrum A., Nur Sabrina H., Arganis L., Saraswati R., Mochtar R. (2018). Application of iota and kappa carrageenans to traditional several food using modified cassava flour. Iop Conf. Ser. Earth Environ. Sci..

[20] Azizi S., Mohamad R., Abdul Rahim R., Mohammadinejad R., Bin Ariff A. (2017). Hydrogel beads bio-nanocomposite based on Kappa-Carrageenan and green synthesized silver nanoparticles for biomedical applications. Int. J. Biol. Macromol..

[21] Deng Y., Huang M., Sun D., Hou Y., Li Y., Dong T., Wang X., Zhang L., Yang W. (2018). Dual Physically Cross-Linked κ-Carrageenan-Based Double Network Hydrogels with Superior Self-Healing Performance for Biomedical Application. ACS Appl. Mater. Interfaces.

[22] Wu Y.-S., Ngai S.-C., Goh B.-H., Chan K.-G., Lee L.-H., Chuah L.-H. (2017). Anticancer Activities of Surfactin and Potential Application of Nanotechnology Assisted Surfactin Delivery. Front. Pharmacol..

[23] Goussous S.A., Casford M.T.L., Murphy A.C., Salmond G.P.C., Leeper F.J., Davies P.B. (2017). Structure of the Fundamental Lipopeptide Surfactin at the Air/Water Interface Investigated by Sum Frequency Generation Spectroscopy. J. Phys. Chem. B.

[24] Horng Y.-B., Yu Y.-H., Dybus A., Hsiao F.S.-H., Cheng Y.-H. (2019). Antibacterial activity of Bacillus species-derived surfactin on Brachyspira hyodysenteriae and Clostridium perfringens. AMB Express.

[25] Bucci A.R., Marcelino L., Mendes R.K., Etchegaray A. (2018). The antimicrobial and antiadhesion activities of micellar solutions of surfactin, CTAB and CPCl with terpinen-4-ol: Applications to control oral pathogens. World J. Microbiol. Biotechnol..

[26] Seydlová G., Svobodová J. (2008). Review of surfactin chemical properties and the potential biomedical applications. Open Med..

[27] Santos V.S.V., Silveira E., Pereira B.B. (2018). Toxicity and applications of surfactin for health and environmental biotechnology. J. Toxicol. Environ. Health Part B.

[28] Lima T.A., Etchegaray A., Machini M.T. (2020). Design, synthesis and valued properties of surfactin oversimplified analogues. Amino Acids.

[29] Sudarmono P., Wibisana A., Listriyani L.W., Sungkar S. (2019). Characterization and Synergistic Antimicrobial Evaluation of Lipopeptides from Bacillus amyloliquefaciens Isolated from Oil-Contaminated Soil. Int. J. Microbiol..

[30] Huang W., Lang Y., Hakeem A., Lei Y., Gan L., Yang X. (2018). Surfactin-based nanoparticles loaded with doxorubicin to overcome multidrug resistance in cancers. Int. J. Nanomed..

[31] Zhang Y., Liu C., Dong B., Ma X., Hou L., Cao X., Wang C. (2015). Anti-inflammatory Activity and Mechanism of Surfactin in Lipopolysaccharide-Activated Macrophages. Inflammation.

[32] Han Y.W. (2015). *Fusobacterium nucleatum*: A commensal-turned pathogen. Curr. Opin. Microbiol.

[33] Brennan C.A., Garrett W.S. (2019). *Fusobacterium nucleatum*—Symbiont, opportunist and oncobacterium. Nat. Rev. Microbiol..

[34] Souto R., Silva-Boghossian C.M., Colombo A.P.V. (2014). Prevalence of *Pseudomonas aeruginosa* and *Acinetobacter* spp. in subgingival biofilm and saliva of subjects with chronic periodontal infection. Braz. J. Microbiol..

[35] Vieira Colombo A.P., Magalhães C.B., Hartenbach F.A.R.R., Martins do Souto R., Maciel da Silva-Boghossian C. (2016). Periodontal-disease-associated biofilm: A reservoir for pathogens of medical importance. Microb. Pathog..

[36] Bansal M., Khatri M., Taneja V. (2013). Potential role of periodontal infection in respiratory diseases—A review. J. Med. Life.

[37] Faure E., Kwong K., Nguyen D. (2018). *Pseudomonas aeruginosa* in Chronic Lung Infections: How to Adapt within the Host?. Front. Immunol.

[38] Ara T., Kurata K., Hirai K., Uchihashi T., Uematsu T., Imamura Y., Furusawa K., Kurihara S., Wang P.-L. (2009). Human gingival fibroblasts are critical in sustaining inflammation in periodontal disease. J. Periodontal. Res..

[39] Hwang Y.Y., Ramalingam K., Bienek D.R., Lee V., You T., Alvarez R. (2013). Antimicrobial activity of nanoemulsion in combination with cetylpyridinium chloride in multidrug-resistant Acinetobacter baumannii. Antimicrob. Agents Chemother..

[40] Kong Z.-L., Johnson A., Ko F.-C., He J.-L., Cheng S.-C. (2018). Effect of Cistanche Tubulosa Extracts on Male Reproductive Function in Streptozotocin⁻Nicotinamide-Induced Diabetic Rats. Nutrients.

[41] Goel S., Mishra P.J.A.M. (2018). Thymoquinone inhibits biofilm formation and has selective antibacterial activity due to ROS generation. Appl. Microbiol. Biotechnol..

[42] van Tonder A., Joubert A.M., Cromarty A.D. (2015). Limitations of the 3-(4,5-dimethylthiazol-2-yl)-2,5-diphenyl-2H-tetrazolium bromide (MTT) assay when compared to three commonly used cell enumeration assays. BMC Res. Notes.

[43] Yonny M.E., García E.M., López A., Arroquy J.I., Nazareno M.A. (2016). Measurement of malondialdehyde as oxidative stress biomarker in goat plasma by HPLC-DAD. Microchem. J..

[44] Ambati M., Rani K.R., Reddy P.V., Suryaprasanna J., Dasari R., Gireddy H. (2017). Evaluation of oxidative stress in chronic periodontitis patients following systemic antioxidant supplementation: A clinical and biochemical study. J. Nat. Sci. Biol. Med..

[45] Kuka G.I., Gursoy H., Emekli-Alturfan E., Ustundag U.V., Kuru B. (2019). Evaluation of nitric oxide levels in chronic periodontitis patients treated with initial periodontal therapy and probiotic food supplements: A double blind, randomized controlled clinical trial. Biotechnol. Biotechnol. Equip..

[46] Nath S.G., Raveendran R. (2013). Microbial dysbiosis in periodontitis. J. Indian Soc. Periodontol..

[47] Hajishengallis G., Sahingur S.E. (2014). Novel inflammatory pathways in periodontitis. Adv. Dent. Res..

[48] Di Benedetto A., Gigante I., Colucci S., Grano M. (2013). Periodontal Disease: Linking the Primary Inflammation to Bone Loss. Clin. Dev. Immunol..

[49] Halib N., Perrone F., Cemazar M., Dapas B., Farra R., Abrami M., Chiarappa G., Forte G., Zanconati F., Pozzato G. (2017). Potential Applications of Nanocellulose-Containing Materials in the Biomedical Field. Materials.

[50] Kalsoom Khan A., Saba A.U., Nawazish S., Akhtar F., Rashid R., Mir S., Nasir B., Iqbal F., Afzal S., Pervaiz F. (2017). Carrageenan Based Bionanocomposites as Drug Delivery Tool with Special Emphasis on the Influence of Ferromagnetic Nanoparticles. Oxidative Med. Cell. Longev..

[51] Meena K.R., Sharma A., Kanwar S.S. (2020). Antitumoral and Antimicrobial Activity of Surfactin Extracted from *Bacillus subtilis* KLP2015. Int. J. Pept. Res. Ther..

[52] Park S.Y., Kim Y.H., Kim E.-K., Ryu E.Y., Lee S.-J. (2010). Heme oxygenase-1 signals are involved in preferential inhibition of pro-inflammatory cytokine release by surfactin in cells activated with *Porphyromonas gingivalis* lipopolysaccharide. Chem. Biol. Interact..

[53] Kim S.D., Cho J.Y., Park H.J., Lim C.R., Lim J.H., Yun H.I., Park S.C., Kim S.K., Rhee M.H. (2006). A Comparison of the anti-inflammatory activity of surfactin A, B, C, and D from *Bacillus subtilis*. J. Microbiol. Biotechnol..

[54] Andrews J.M. (2001). Determination of minimum inhibitory concentrations. J. Antimicrob. Chemother..

[55] Balouiri M., Sadiki M., Ibnsouda S.K. (2016). Methods for in vitro evaluating antimicrobial activity: A review. J. Pharm. Anal..

[56] Veloo A.C.M., Seme K., Raangs E., Rurenga P., Singadji Z., Wekema-Mulder G., van Winkelhoff A.J. (2012). Antibiotic susceptibility profiles of oral pathogens. Int. J. Antimicrob. Agents.

[57] Kulik E.M., Lenkeit K., Chenaux S., Meyer J. (2008). Antimicrobial susceptibility of periodontopathogenic bacteria. J. Antimicrob. Chemother..

[58] Mustafa M.-H., Chalhoub H., Denis O., Deplano A., Vergison A., Rodriguez-Villalobos H., Tunney M.M., Elborn J.S., Kahl B.C., Traore H. (2016). Antimicrobial Susceptibility of *Pseudomonas aeruginosa* Isolated from Cystic Fibrosis Patients in Northern Europe. Antimicrob. Agents Chemother..

[59] Reller L.B., Weinstein M., Jorgensen J.H., Ferraro M.J. (2009). Antimicrobial Susceptibility Testing: A Review of General Principles and Contemporary Practices. Clin. Infect. Dis..

[60] Hamouda T., Baker J.R. (2000). Antimicrobial mechanism of action of surfactant lipid preparations in enteric Gram-negative bacilli. J. Appl. Microbiol..

[61] Zhou Z., Liu F., Zhang X., Zhou X., Zhong Z., Su H., Li J., Li H., Feng F., Lan J. (2018). Cellulose-dependent expression and antibacterial characteristics of surfactin from *Bacillus subtilis* HH2 isolated from the giant panda. PLoS ONE.

[62] Mercado N., Bhatt P., Sutariya V., Florez F.L.E., Pathak Y.V., Pathak Y.V. (2019). Application of Nanoparticles in Treating Periodontitis: Preclinical and Clinical Overview. Surface Modification of Nanoparticles for Targeted Drug Delivery.

[63] Zorraquín-Peña I., Cueva C., González de Llano D., Bartolomé B., Moreno-Arribas M.V. (2020). Glutathione-Stabilized Silver Nanoparticles: Antibacterial Activity against Periodontal Bacteria, and Cytotoxicity and Inflammatory Response in Oral Cells. Biomedicines.

[64] Yu O.Y., Zhao I.S., Mei M.L., Lo E.C.-M., Chu C.-H. (2017). Dental Biofilm and Laboratory Microbial Culture Models for Cariology Research. Dent. J..

[65] Mira A., Buetas E., Rosier B.T., Mazurel D., Villanueva-Castellote Á., Llena C., Ferrer M.D. (2019). Development of an in vitro system to study oral biofilms in real time through impedance technology: Validation and potential applications. J. Oral Microbiol..

[66] Chandki R., Banthia P., Banthia R. (2011). Biofilms: A microbial home. J. Indian Soc. Periodontol..

[67] Meena K.R., Kanwar S.S. (2015). Lipopeptides as the antifungal and antibacterial agents: Applications in food safety and therapeutics. Biomed. Res. Int..

[68] Rivardo F., Turner R.J., Allegrone G., Ceri H., Martinotti M.G. (2009). Anti-adhesion activity of two biosurfactants produced by *Bacillus* spp. prevents biofilm formation of human bacterial pathogens. Appl. Microbiol. Biotechnol..

[69] Moryl M., Spętana M., Dziubek K., Paraszkiewicz K., Różalska S., Płaza G.A., Różalski A. (2015). Antimicrobial, antiadhesive and antibiofilm potential of lipopeptides synthesised by *Bacillus subtilis*, on uropathogenic bacteria. Acta Biochim. Pol..

[70] Benov L. (2019). Effect of growth media on the MTT colorimetric assay in bacteria. PLoS ONE.

[71] Damas-Souza D.M., Nunes R., Carvalho H.F. (2019). An improved acridine orange staining of DNA/RNA. Acta Histochem..

[72] Plemel J.R., Caprariello A.V., Keough M.B., Henry T.J., Tsutsui S., Chu T.H., Schenk G.J., Klaver R., Yong V.W., Stys P.K. (2017). Unique spectral signatures of the nucleic acid dye acridine orange can distinguish cell death by apoptosis and necroptosis. J. Cell Biol..

[73] Liu T.-H., Tsai T.-Y., Pan T.-M. (2018). Effects of an ethanol extract from Lactobacillus paracasei subsp. paracasei NTU 101 fermented skimmed milk on lipopolysaccharide-induced periodontal inflammation in rats. Food Funct..

[74] Wang F., Yao Z., Wu H., Zhang S., Zhu N., Gai X. (2011). Antibacterial Activities of Kappa-Carrageenan Oligosaccharides. Applied Mechanics and Materials.

[75] Soares A.S.L.S., Scelza M.Z., Spoladore J., Gallito M.A., Oliveira F., Moraes R.C.M., Alves G.G. (2018). Comparison of primary human gingival fibroblasts from an older and a young donor on the evaluation of cytotoxicity of denture adhesives. J. Appl. Oral Sci..

[76] Dahiya P., Kamal R., Gupta R., Bhardwaj R., Chaudhary K., Kaur S. (2013). Reactive oxygen species in periodontitis. J. Indian Soc. Periodontol..

[77] D’Aiuto F., Nibali L., Parkar M., Patel K., Suvan J., Donos N. (2010). Oxidative stress, systemic inflammation, and severe periodontitis. J. Dent. Res..

[78] Mittal M., Siddiqui M.R., Tran K., Reddy S.P., Malik A.B. (2014). Reactive oxygen species in inflammation and tissue injury. Antioxid Redox Signal..

[79] Liu C., Mo L., Niu Y., Li X., Zhou X., Xu X. (2017). The Role of Reactive Oxygen Species and Autophagy in Periodontitis and Their Potential Linkage. Front. Physiol..

[80] Wang Y., Huang X., He F. (2019). Mechanism and role of nitric oxide signaling in periodontitis. Exp. Med..

[81] Esfandiari N., Sharma R.K., Saleh R.A., Thomas A.J., Agarwal A. (2003). Utility of the nitroblue tetrazolium reduction test for assessment of reactive oxygen species production by seminal leukocytes and spermatozoa. J. Androl..

[82] Nita M., Grzybowski A. (2016). The Role of the Reactive Oxygen Species and Oxidative Stress in the Pathomechanism of the Age-Related Ocular Diseases and Other Pathologies of the Anterior and Posterior Eye Segments in Adults. Oxidative Med. Cell. Longev..

[83] Grotto D., Maria L., Valentini J., Paniz C., Schmitt G., Garcia S., Pomblum V., Rocha J.B., Farina M. (2009). Importance of the lipid peroxidation biomarkers and methodological aspects FOR malondialdehyde quantification. Quim. Nova Quim Nova.

[84] Yonny M.E., Rodríguez Torressi A., Nazareno M.A., Cerutti S. (2017). Development of a Novel, Sensitive, Selective, and Fast Methodology to Determine Malondialdehyde in Leaves of Melon Plants by Ultra-High-Performance Liquid Chromatography-Tandem Mass Spectrometry. J. Anal. Methods Chem.

[85] Ambili R., Janam P. (2017). A critique on nuclear factor-kappa B and signal transducer and activator of transcription 3: The key transcription factors in periodontal pathogenesis. J. Indian Soc. Periodontol..

[86] Yucel-Lindberg T., Båge T. (2013). Inflammatory mediators in the pathogenesis of periodontitis. Expert Rev. Mol. Med..

[87] Grover H., Saini R., Bhardwaj P., Bhardwaj A. (2016). Cytokines and Other Inflammatory Mediators in Periodontal Health and Disease. Indian J. Oral Health Res..

[88] Osorio R., Alfonso-Rodríguez C.A., Medina-Castillo A.L., Alaminos M., Toledano M. (2016). Bioactive Polymeric Nanoparticles for Periodontal Therapy. PLoS ONE.

[89] Martin V., Ribeiro I.A.C., Alves M.M., Gonçalves L., Almeida A.J., Grenho L., Fernandes M.H., Santos C.F., Gomes P.S., Bettencourt A.F. (2019). Understanding intracellular trafficking and anti-inflammatory effects of minocycline chitosan-nanoparticles in human gingival fibroblasts for periodontal disease treatment. Int. J. Pharm..

